# Supplementary material for: Cystatin B is essential for proliferation and interneuron migration in individuals with EPM1 epilepsy
Source: EMBO Mol Med. 2020 May 7;12(6):e11419. doi: 10.15252/emmm.201911419 (PMC7278547; doi:10.15252/emmm.201911419)
Supplement: Supplementary file 1 — Appendix [file EMMM-12-e11419-s001.pdf]

Appendix

Cystatin B is essential for proliferation and interneuron migration in individuals with EPM1 epilepsy

Francesco Di Matteo[1,2,7], Fabrizia Pipicelli[1,2,7], Christina Kyrousi[1], Isabella Tovecci[1,3], Eduardo Penna[3], Marianna Crispino[3], Angela Chambery[4], Rosita Russo[4], Ane Cristina Ayo-Martin[1,2], Martina Giordano[1], Anke Hoffmann[1], Emilio Ciusani[5], Laura Canafoglia[5], Magdalena Götz[6], Rossella Di Giaimo[1,3,*] and Silvia Cappello[1,*].

Table Of Content:

Appendix Table S1 (exact p-values for each figures)

Appendix table S1

Content: Exact p-values for each panel in the figures.

Figure 1.

A.

16 vs 23	P=0.0232
23 vs 50	P=0.0026

C.

PAX6	P=0.0062
NEUN	P=0.0213
NESTIN	P=0.0327

Figure2.

B.

CTRL vs CSTB	P=0.0286

D.

CTRL vs Cstb	P=0.0286

E.

CTRL vs Cstb	P=0.0286

H.

Bin3	P=0.0286

I.

Bin1	P=0.0221
Bin3	P=0.0012

J.

Bin3	P=0.0159
Bin4	P=0.0159

K.

Bin3	P=0.0200

Figure 3.

E.

Bin3	P=0.0286

G.

Bin3	P=0.0286

Figure 4.

B.

Bin2	P=0.0286
Bin4	P=0.0286
Bin5	P=0.0286

D.

CTRL vs KD	P=0.0286

F.

CTRL vs R68X	P<0.0001
CTRL vs CSTB	P=0.0008
R68X vs CSTB	P=0.0003

G.

CTRL vs R68X	P=0.0265

I.

Bin1	P=0.0286
Bin2	P=0.0286
Bin3	P=0.0286

J.

Bin1	P=0.0167
Bin2	P=0.0167
Bin3	P=0.0167

K.

R68X vs CSTB	P=0.0061

M.

R68X vs Cstb	P=0.0074

O.

R68X vs CSTB	P=0.0114

Figure 5.

A.

d4	f-CTRL vs UL1	P<0.001
d4	f-CTRL vs UL4	P<0.001
d4	b-CTRL vs UL1	P<0.001
d4	b-CTRL vs UL4	P=0.0479
d7	f-CTRL vs UL1	P<0.001
d7	f-CTRL vs UL4	P<0.001
d7	b-CTRL vs UL1	P<0.001
d7	b-CTRL vs UL1	P<0.001
d9	f-CTRL vs UL1	P<0.001
d9	f-CTRL vs UL4	P<0.001
d9	b-CTRL vs UL1	P<0.001

d9	b-CTRL vs UL1	P<0.001
d11	f-CTRL vs UL1	P<0.001
d11	f-CTRL vs UL4	P<0.001
d11	b-CTRL vs UL1	P<0.001
d11	b-CTRL vs UL1	P<0.001

C.

CTRL vs UL1	P=0.0002

D.

CTRL vs UL1	P=0.0005

F.

CTRL vs UL1	P=0.0001
CTRL vs UL4	P=0.0001

G.

CTRL vs UL1	P=0.0005
CTRL vs UL4	P=0.0005

H.

CTRL-CTRLmedium vs CTRL-UL4medium	P=0.0237
UL4-UL4medium vs UL4-CTRLmedium	P=0.0286
CTRL-CTRLmedium vs UL4-UL4medium	P=0.0286

Figure 6.

B.

CTRL vs UL1	P<0.0001
CTRL vs UL4	P<0.0001

D.

CTRL vs UL1	P<0.0001
CTRL vs UL4	P<0.0001

E.

CTRL vs UL1	P=0.0286
CTRL vs UL4	P=0.0286

H.

Dorsal f-CTRL vs dorsal UL1	P=0.0426

Figure EV2.

K.

CTRL vs Cstb	P=0.0125

Figure EV3.

M.

Cstb vs R68X	P=0.0003
R68X vs KD	P=0.0048

Figure EV4.

B.

f-CTRL vs UL1	P=0.0065
f-CTRL vs UL4	P=0.0013
b-CTRL vs UL1	P=0.0018
b-CTRL vs UL4	P=0.0018

E.

CTRL vs UL1	P=0.0045
CLTR vs UL4	P=0.0022

F.

CTRL vs UL1	P=0.0010
CLTR vs UL4	P=0.0006

I.

CTRL-CTRLmedium vs CTRL-UL4medium	P=0.0258
CTRL-CTRLmedium vs UL4-UL4medium	P=0.0064
CTRL-CTRLmedium vs UL4-CTRLmedium	P=0.0146

Figure EV5.

C.

CTRL vs UL1	P=0.0022
CTRL vs UL4	P=0.0022

D.

CTRL vs UL1	P=0.0014
CTRL vs UL4	P=0.0014
