# Supplementary material for: Cystatin B is essential for proliferation and interneuron migration in individuals with EPM1 epilepsy
Source: EMBO Mol Med. 2020 May 7;12(6):e11419. doi: 10.15252/emmm.201911419 (PMC7278547; doi:10.15252/emmm.201911419)
Supplement: Supplementary file 2 — Expanded View Figures PDF [file EMMM-12-e11419-s002.pdf]

Expanded View Figures

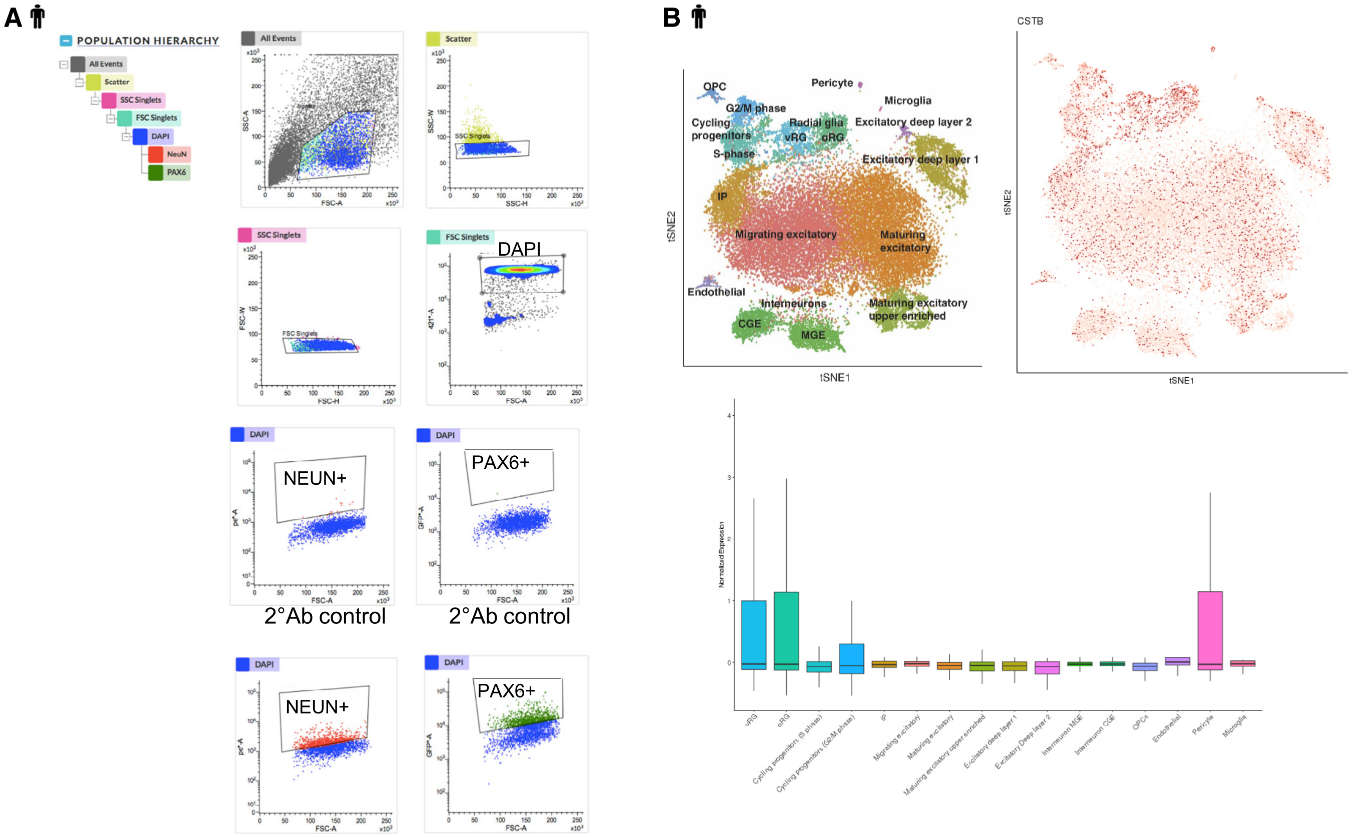

Figure EV1. CSTB expression during cortical development in hCOs and human tissue.

A FACS strategy for nuclei sorting from d135 hCOs. NEUN antibody was used to select neuronal nuclei and PAX6 antibody to select progenitor cells. Control plots obtained with secondary antibodies (2° Ab) only are also shown.

B Top: tSNE plots colored by cell types isolated from human embryonic cortical tissues on the left and by *CSTB* gene expression, red, on the right. Bottom: Boxplot of *CSTB* relative expression in each of the cell types isolated from human embryonic cortical tissues (Polioudakis *et al*, 2019).

© 2020 The Authors

Figure EV2. CSTB overexpression induces progenitor cell expansion in hCOs and in the developing mouse cortex.

A Quantification of the total number of transfected proliferating $KI67^{+}GFP^{+}$ cells/area (μm^2) of ventricle-like structures of d40 hCOs electroporated with GFP-empty vector control or GFP-Cstb and analyzed 5 dpe. Data shown as *Z*-scores relative to the mean of b-CTRL.

B, C Quantification of the total number of transfected proliferating $Ki67^{+}GFP^{+}$ cells/area (μm^2) (B) and $PH3^{+}$ cells/area (μm^2) (C) of ventricular structures of E16 mouse cerebral cortices electroporated at E14 with GFP-empty vector or Cstb and analyzed 2 dpe.

D Micrograph of coronal sections of E16 mouse cerebral cortices electroporated at E14 with GFP-empty vector or Cstb, analyzed 2 dpe, and immunostained with GFP and Ccnd1. Ventricle (V) is indicated. The dashed lines represent the apical surface of the ventricles.

E Quantification of the total number of proliferating $Ccnd1^{+}$ cells/area (μm^2) of ventricular structures of E16 mouse cerebral cortices electroporated at E14 with GFP-empty vector or Cstb and analyzed 2 dpe in (D).

F–H Quantification of the total number of progenitors $Pax6^{+}$ cells/area (μm^2) (F), intermediate progenitors $Tbr2^{+}$ cells/area (μm^2) (G) and $Pax6^{+}Tbr2^{+}$ cells/area (μm^2) (H) of ventricular structures of E16 mouse cerebral cortices electroporated at E14 with GFP-empty vector or Cstb and analyzed 2 dpe. Data shown as *Z*-scores relative to the mean of CTRL.

I, J Distribution of electroporated $Pax6^{+}$ cells (I) and progenitor GFP^{+} cells (J) in the mouse cortex. The cortex was subdivided into 5 equal bins—Bin1 corresponded to the apical side and Bin5 to the pial side of the cortex and GFP^{+} cells or $Pax6^{+}$ cells calculated in each Bin.

K Quantification of the total number of non-transfected proliferating $PH3^{+}$ GFP negative cells/area of Bin3 out of the 5 equally distributed 5 bins in E16 mouse cerebral cortices electroporated at E14 with GFP-empty vector or Cstb and analyzed 2 dpe.

Data information: Scale bars: 100 μm in (D). Data are represented as mean ± SEM. Statistical significance was based on Mann–Whitney test (*$P < 0.05$, ns = not significant according to the test). Every dot in the plots refers to independent analyzed ventricles per hCO from at least 3 different hCOs generated in at least 2 independent batches, or independent analyzed mouse brains. Exact *P*-values in Appendix Table S1.
Source data are available online for this figure.

© 2020 The Authors

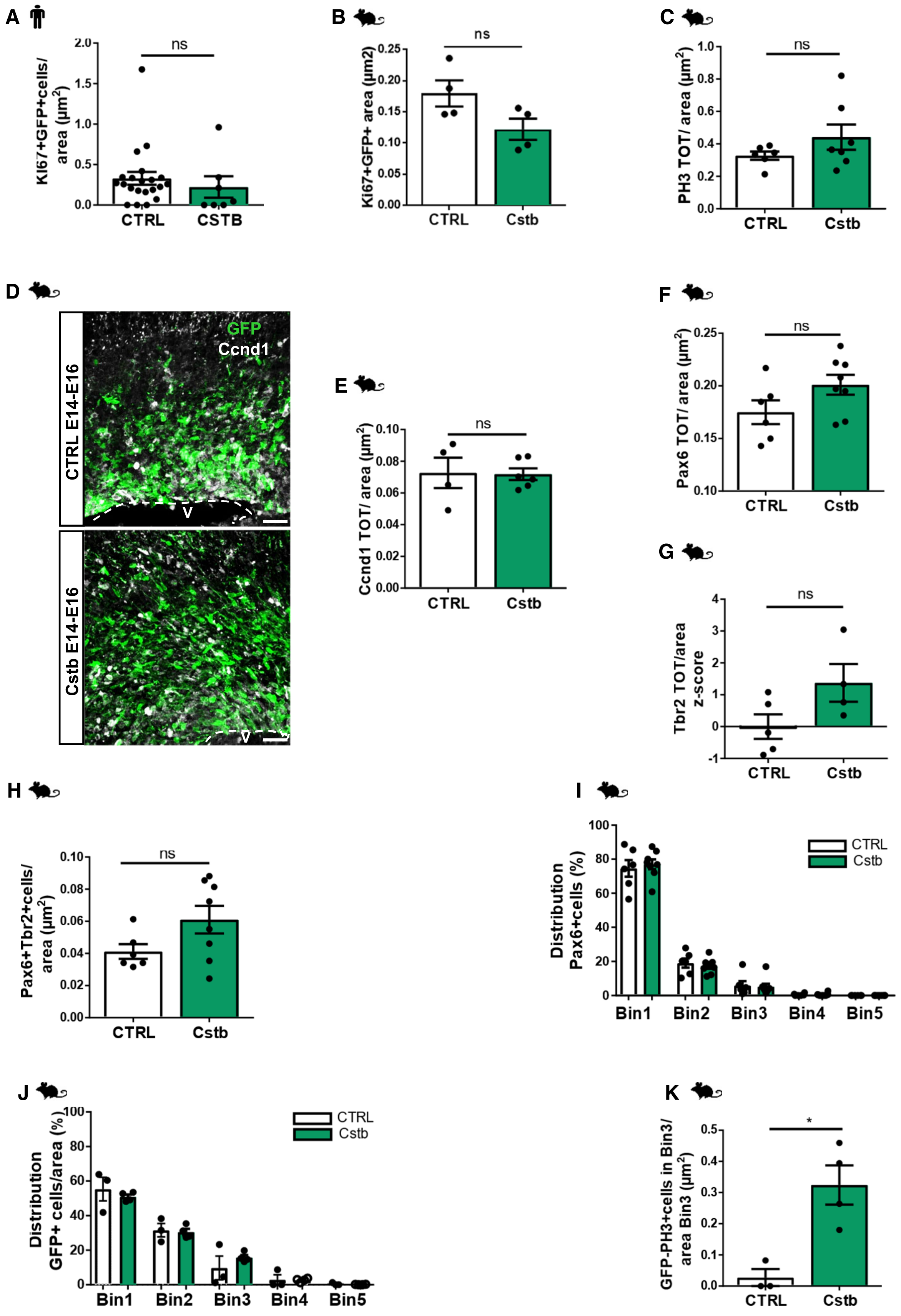

Figure EV2.

 © 2020 The Authors

Figure EV3. CSTB is secreted and induces recruitment of migrating interneurons. Downregulation of Cstb and R68X overexpression results in a decreased number of progenitors and migrating interneurons in the developing mouse cortex.

A Western blot analysis for Cstb in the CM from E14 cortical cells in culture for 4 days. The primary cells were transfected with a plasmid expressing GFP alone or GFP-Cstb. In the lanes of the CMs derived from cells transfected with GFP as control, only a band is detectable corresponding to the endogenous Cstb; in the lanes of the CMs derived from cells transfected with GFP-Cstb, one band corresponding to the endogenous Cstb and another one corresponding to the exogenous GFP-Cstb are detectable.

B Western blot analysis for GFP in the CM from E14 cortical cells in culture for 2, 4, and 7 days. The primary cells were transfected with a plasmid expressing GFP alone or GFP-Cstb. In the lanes of the CMs derived from cells transfected with GFP as control, no band is detectable; in the lanes of the CMs derived from cells transfected with GFP-Cstb, one band corresponding to the fused GFP-Cstb is detectable.

C Western blot analysis for Cstb in the CM from E14 cortical cells in culture for 4 days. Top lane: CM from cells treated with nocodazole and untreated control cells. Middle lane: CM from cells transfected with control plasmid and *Kif1a*-shRNA. Bottom lane: CM from cells transfected with control plasmid and *Kif5a*-shRNA.

D Quantification of WB in C.

E Quantification of the total number of GAD67-GFP interneurons/area (μm^2), related to Fig 3F.

F Quantification of the total number of GAD65-GFP interneurons/area, related to Fig 3D.

G Detail of micrograph of coronal sections of E17 cerebral cortices, from GAD67-GFP mice, electroporated at E14, co-electroporated with mCherry-expressing vector and HA-empty vector or HA-Cstb, and analyzed 3 dpe. Immunostaining with RFP to identify electroporated cells and GFP to identify migrating interneurons in the GAD65-GFP transgenic mouse line.

H Immunostaining for GFP (Green, to identify miR-transfected cells), Cstb (red), and DAPI (blue) on primary E14 mouse cortical cells transfected with miRNA-*Cstb*- or miRneg-expressing plasmids and immunostained 3 dpt and 7 dpt.

I Quantification of the total number of transfected proliferating Ki67$^+$ GFP cells/area (μm^2) of ventricular structures transfected with GFP-miR-neg vector (CTRL) or GFP-miRNA-*Cstb* (KD).

J Western blot analysis for Cstb in the CM from E14 cortical cells in culture for 4 days. The primary cells were transfected with a plasmid expressing GFP-R68X mutant Cstb. Only a band is detectable corresponding to the endogenous Cstb; no band corresponding to GFP-R68X is detectable indicating that it is not present in the CM.

K Western blot analysis for Cstb on the protein extracts from E14 primary cortex cells transfected with GFP-Cstb- or GFP-R68X-expressing plasmids. Cstb$^+$ bands corresponding to monomeric and dimeric forms are identified.

L Micrograph of coronal sections of E17 mouse cerebral cortices electroporated at E14 with GFP-Cstb, R68X, or miRNA (KD), analyzed 3 dpe, and immunostained with Dcx.

M Quantification of the total number of ventricles with apically located Dcx$^+$ cells in (L).

Data information: Nuclei (blue) are stained with DAPI. Scale bars: 50 μm in (G and L), 20 μm in (H). Data are represented as mean ± SEM. Statistical significance was based on Mann–Whitney test (*$P < 0.05$, **$P < 0.01$, ns = not significant according to the test) in (D–F) and on exact binomial test (***$P < 0.001$, ****$P < 0.0001$) in (I and M). Every dot in the plots refers to independent analyzed mouse brains. Exact *P*-values in Appendix Table S1.
Source data are available online for this figure.

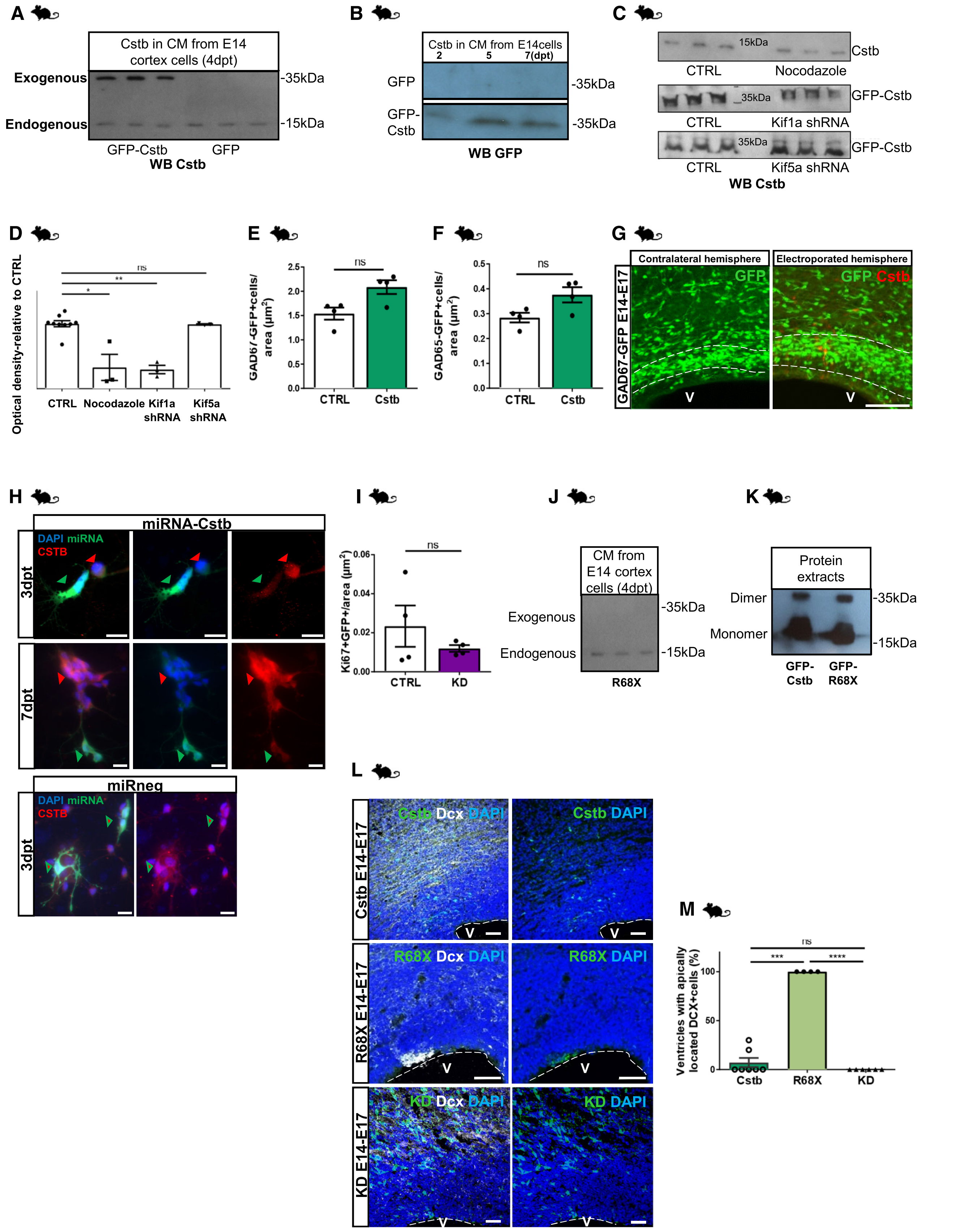

Figure EV3.

© 2020 The Authors

Figure EV4. Proliferation is reduced in EPM1-derived cerebral organoids in a cell-non-autonomous manner.

A Scheme of the reprogramming of control and patient-derived PBMCs to generate hCO. Representative pictures of the reprogramming procedure and hCO generation.

B qRT–PCR results of *CSTB* mRNA expression level in reprogrammed iPSCs from f-CTRL, b-CTRL, UL1, and UL4 PBMCs. Data are represented as mean ± SEM. Statistical significance was based on Student's *t*-test (**$P < 0.01$). Three samples were analyzed as biological replicates

C Western blot analysis for CSTB in the protein extracts from reprogrammed iPSCs from f-CTRL, b-CTRL, UL1, and UL4 PBMCs. GAPDH immunostaining was used as a positive control. Ponceau Red staining is shown to indicate the presence of proteins at comparable levels in the protein extracts.

D Micrographs of 2D cultures from d30 and d60 dissociated b-CTRL, UL1, and UL4 hCOs. Cells were immunostained for KI67.

E, F Quantification of the number of $KI67^+$ cells related to the number of $DAPI^+$ cells in 2D cultures derived from dissociation of d30 (E) and d60 (F). Data are shown as a *Z*-score of b-CTRL and they are represented as mean ± SEM. Statistical significance was based on Mann–Whitney test (**$P < 0.01$, ***$P < 0.001$).

G, H FACS plots of the $Ki67^+$ (G) and $PH3^+$ (H) cells by FACS analysis on 36 days old dissociated hCOs.

I Quantification of the $PH3^+$ cells by FACS analysis on 36 days old dissociated hCOs after exchange of conditioned medium between b-CTRL and UL4 hCOs. Data are represented as mean ± SEM. Statistical significance was based on Mann–Whitney test Statistical significance was based on Mann–Whitney test (*$P < 0.05$, **$P < 0.01$) Four samples were analyzed as biological replicates FACS plots in Fig EV4H.

Data information: Scale bars: 200 μm in (A) and 50 μm in (D). Every dot in the plots refers to the number of quantified independent wells of plated cells derived from a pool of 2 or 3 independent organoids. Exact *P*-values in Appendix Table S1.
Source data are available online for this figure.

© 2020 The Authors

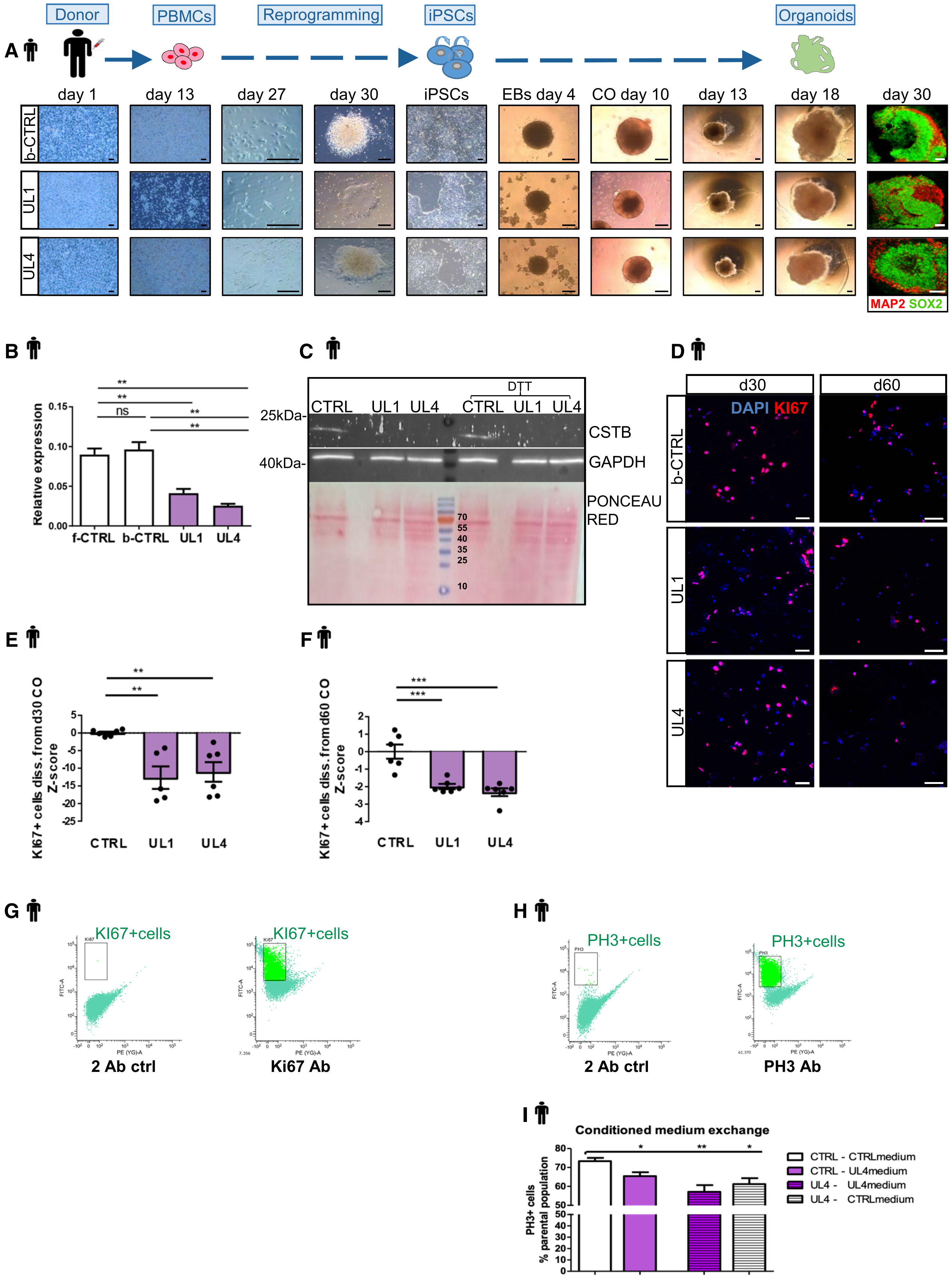

Figure EV4.

 © 2020 The Authors

Figure EV5. EPM1-derived cerebral organoids exhibit premature differentiation. Proteomic analysis in patients' hCOs.

A FACS plots of DCX^+ cells sorted from dissociated d60 hCOs (related to Fig 6E).

B Micrographs of 2D cultures from d30 and d60 dissociated b-CTRL, UL1, and UL4 hCOs. Cells were immunostained for TUBB3.

C, D Quantification of the number of $TUBB3^+$ cells related to the number of $DAPI^+$ cells in 2D cultures derived from dissociation of d30 (C) and d60 (D).

E Western blot analysis of differentially expressed proteins identified by Mass Spectrometry analysis on b-CTRL and UL1 hCOs. Different proteins have been quantified on protein extracts from hCOs from both patient's lines UL1 and UL4 and from b-CTRL samples. Quantifications from three independent samples for CTRL and UL4 and four independent samples for UL1 are shown in the plot as average value ± SEM. Fold changes from proteomic analysis for each protein are reported below the plot.

F The 178 differentially expressed proteins were cross-referenced with the dataset of single-cell RNA-seq from human brain fetal cortex (http://solo.bmap.ucla.edu/shiny/webapp/; Polioudakis *et al*, 2019) to understand which cells express the regulated proteins. They were then categorized according to their cell expression in 4 groups.

G GOChord plot showing relationships between selected representative biological process GO terms and differentially expressed proteins mainly expressed in radial glial cells. The representation shows up (red)- and downregulated (green) proteins differentially expressed in UL1 relative to b-CTRL ($-0.3 \geq \log_2 FC \geq 0.3$).

Data information: Data are shown as a *Z*-score of b-CTRL, and they are represented as mean ± SEM. Statistical significance was based on Mann–Whitney test (**$P < 0.01$). Scale bars: 50 μm in (B). Every dot in the plots refers to the number of quantified independent wells of plated cells derived from a pool of 2 or 3 independent organoids. Exact *P*-values in Appendix Table S1.
Source data are available online for this figure.

© 2020 The Authors

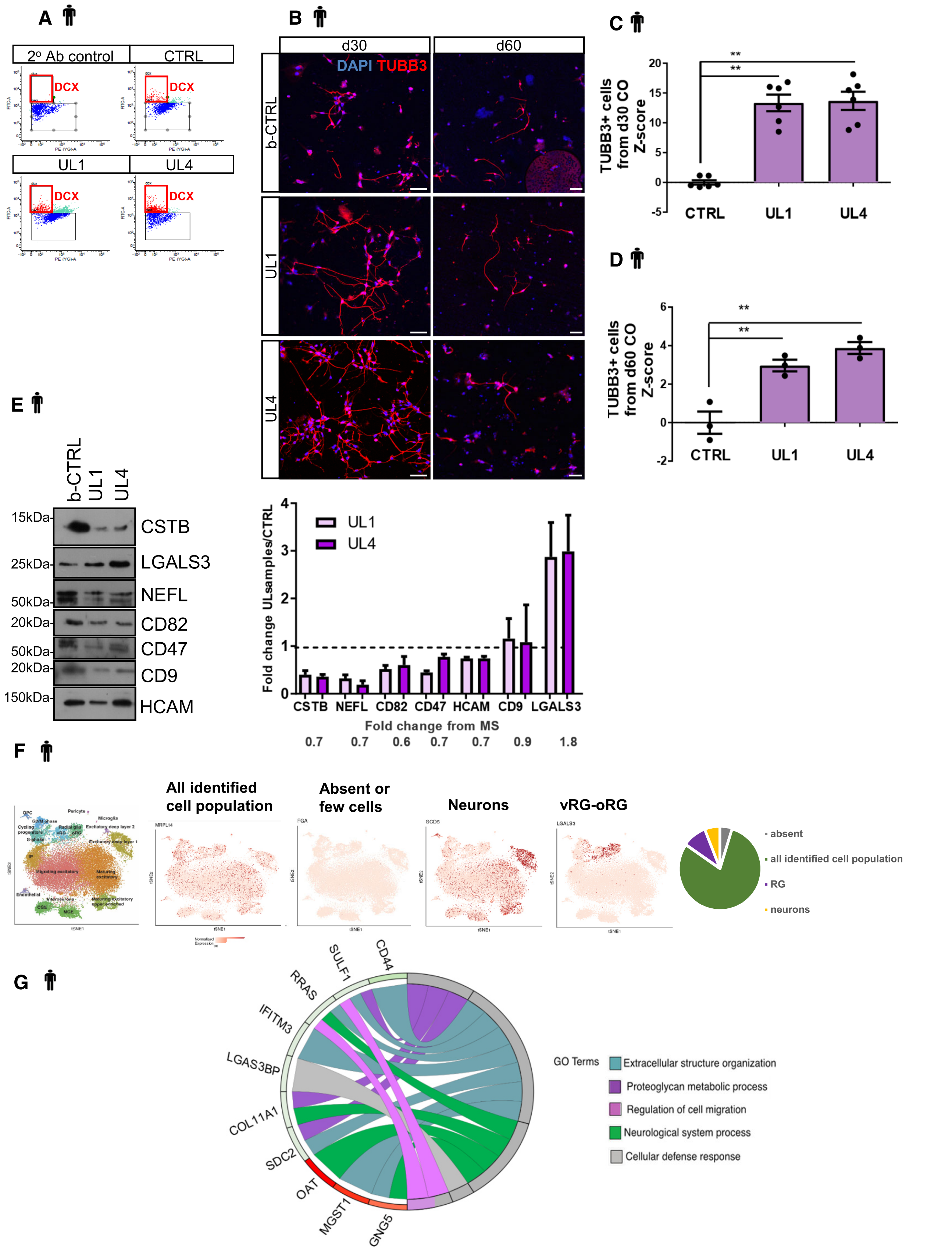

Figure EV5.

 © 2020 The Authors
