# Supplementary material for: Cystatin B is essential for proliferation and interneuron migration in individuals with EPM1 epilepsy
Source: EMBO Mol Med. 2020 May 7;12(6):e11419. doi: 10.15252/emmm.201911419 (PMC7278547; doi:10.15252/emmm.201911419)
Supplement: Supplementary file 5 — Source Data for Expanded View [file EMMM-12-e11419-s011.zip › FigEV5_sourcedata_01042020.pdf]

Figure EV5

Figure EV5B

DAPI TUBB3

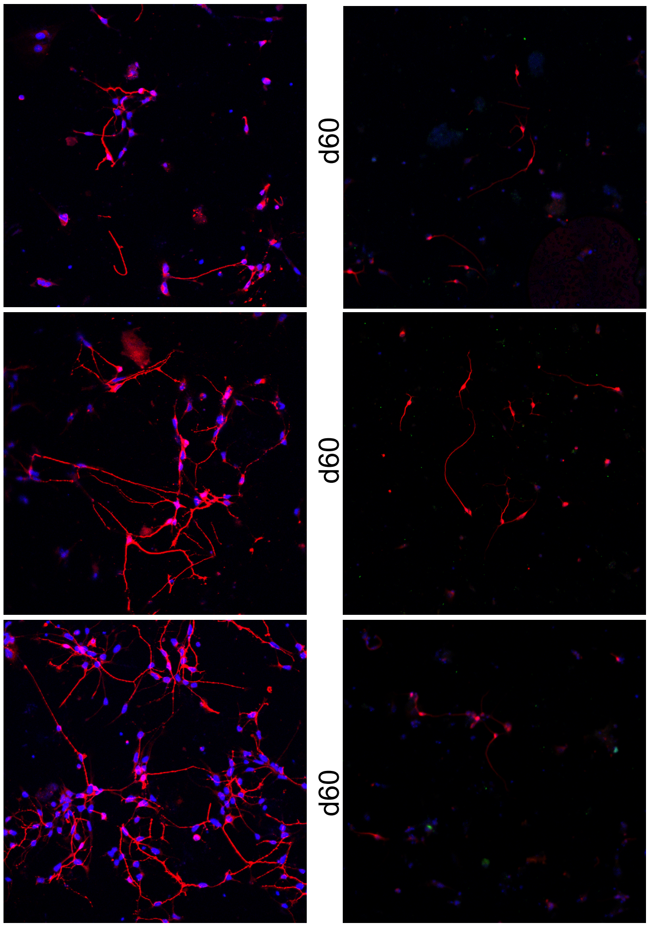

Figure EV5E

UL4 UL1 b-CTRL

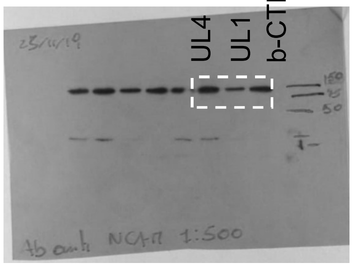

HCAM

UL4 UL1 b-CTRL

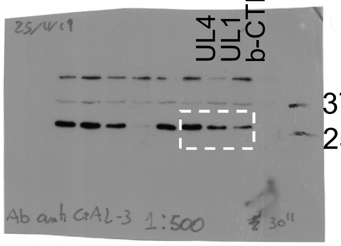

37
25
LGALS3

UL4 UL1 b-CTRL

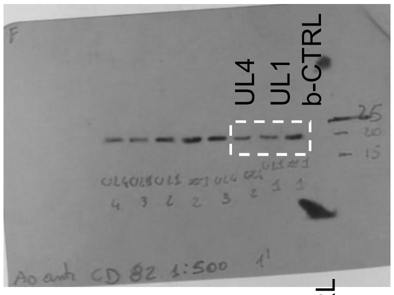

CD82

UL4 UL1 b-CTRL

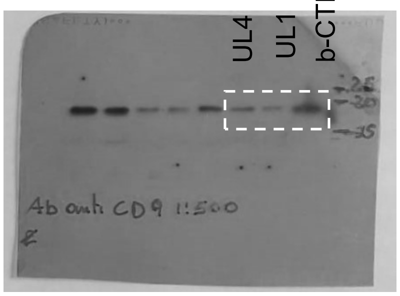

CD9

UL4 UL1 b-CTRL

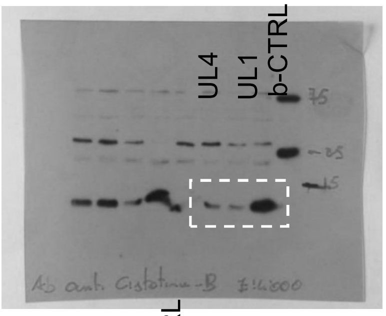

CSTB

Figure EV5E

UL4 UL1 b-CTRL

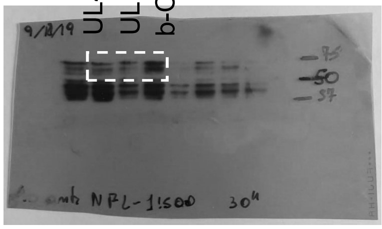

NEFL

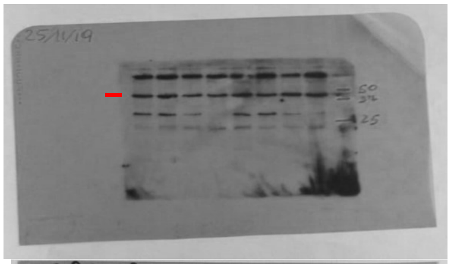

β-actin

UL4 UL1 b-CTRL

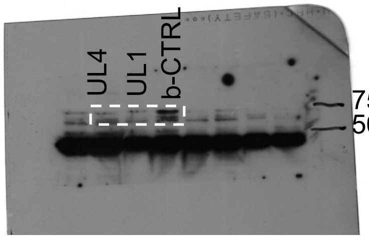

75
50
CD47

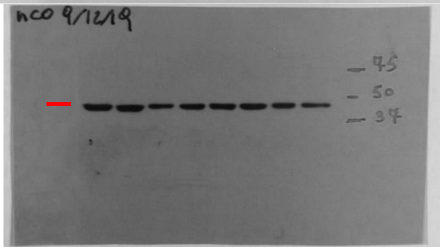

β-actin
