# Supplementary figures and images for: Cystatin B is essential for proliferation and interneuron migration in individuals with EPM1 epilepsy
Source: EMBO Mol Med. 2020 May 7;12(6):e11419. doi: 10.15252/emmm.201911419 (PMC7278547; doi:10.15252/emmm.201911419)

Figure EV3

Figure EV3A-EV3J

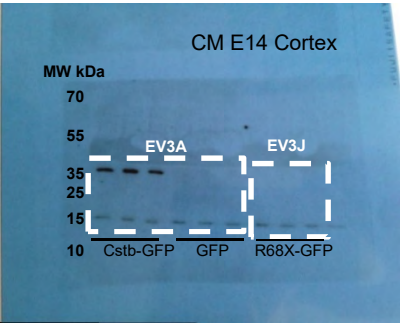

Figure EV3B

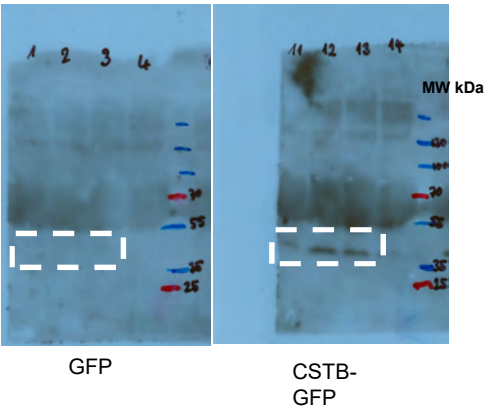

Figure EV3C

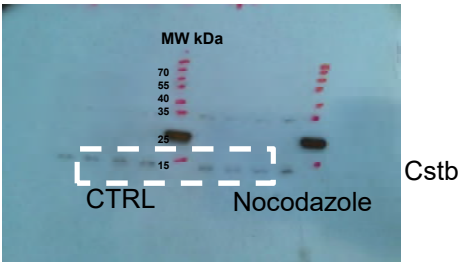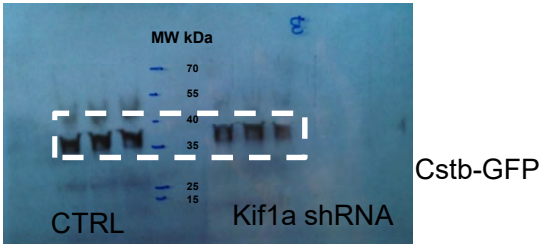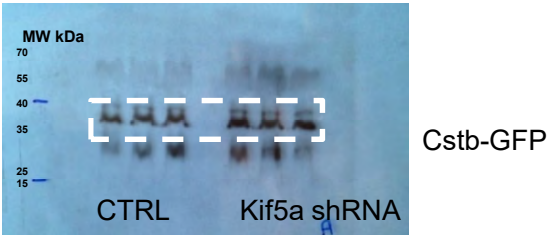

Figure EV3H

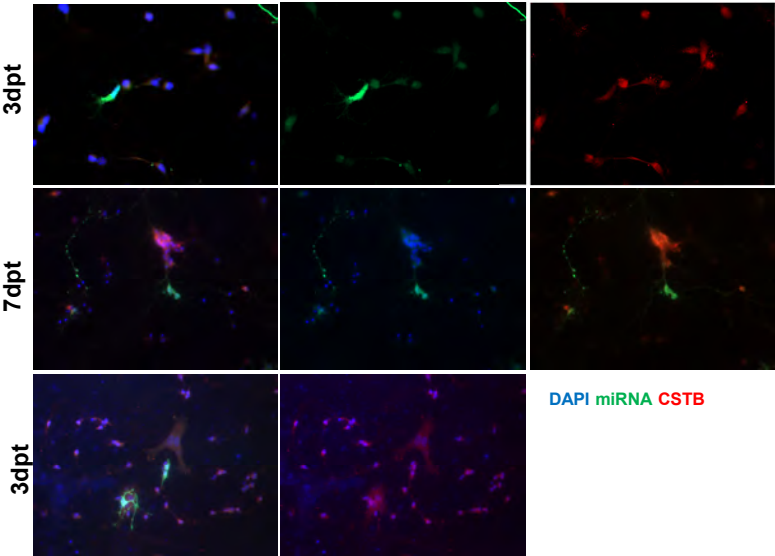

Figure EV3K

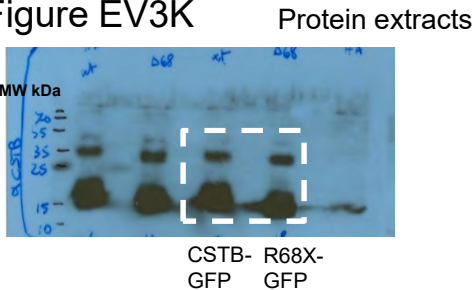

Figure EV3L

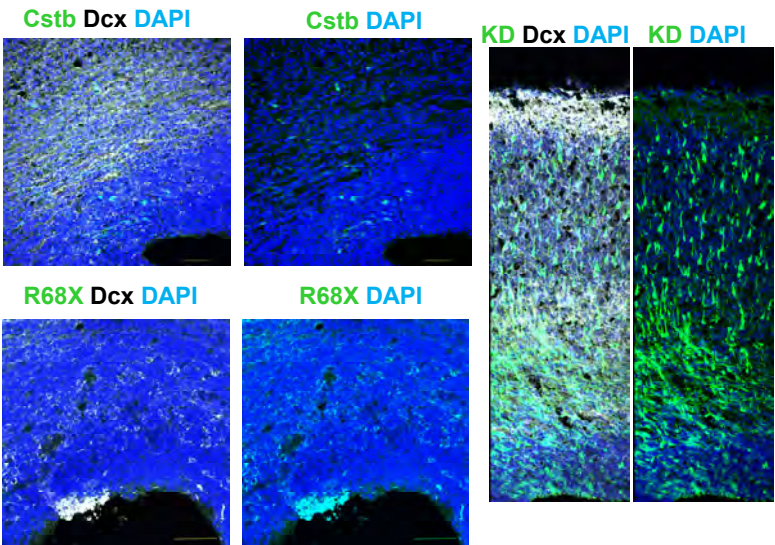

Supplement: Supplementary file 5 — Source Data for Expanded View [file EMMM-12-e11419-s011.zip › FigEV3_sourcedata.pdf]

Figure EV4

Figure EV4C

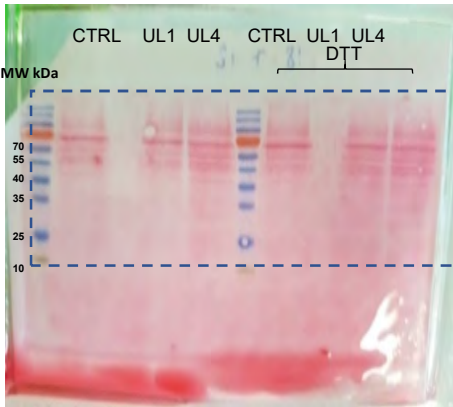

PONCEAU RED

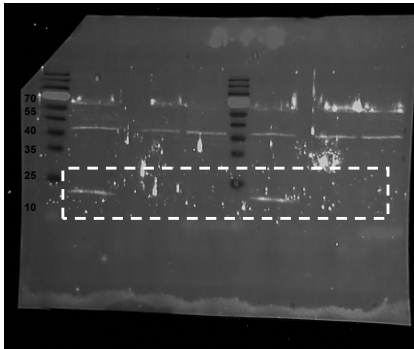

CSTB

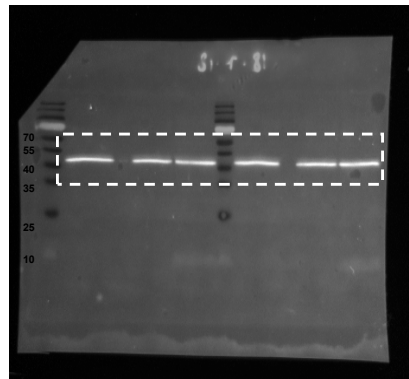

GAPDH

Figure EV4D

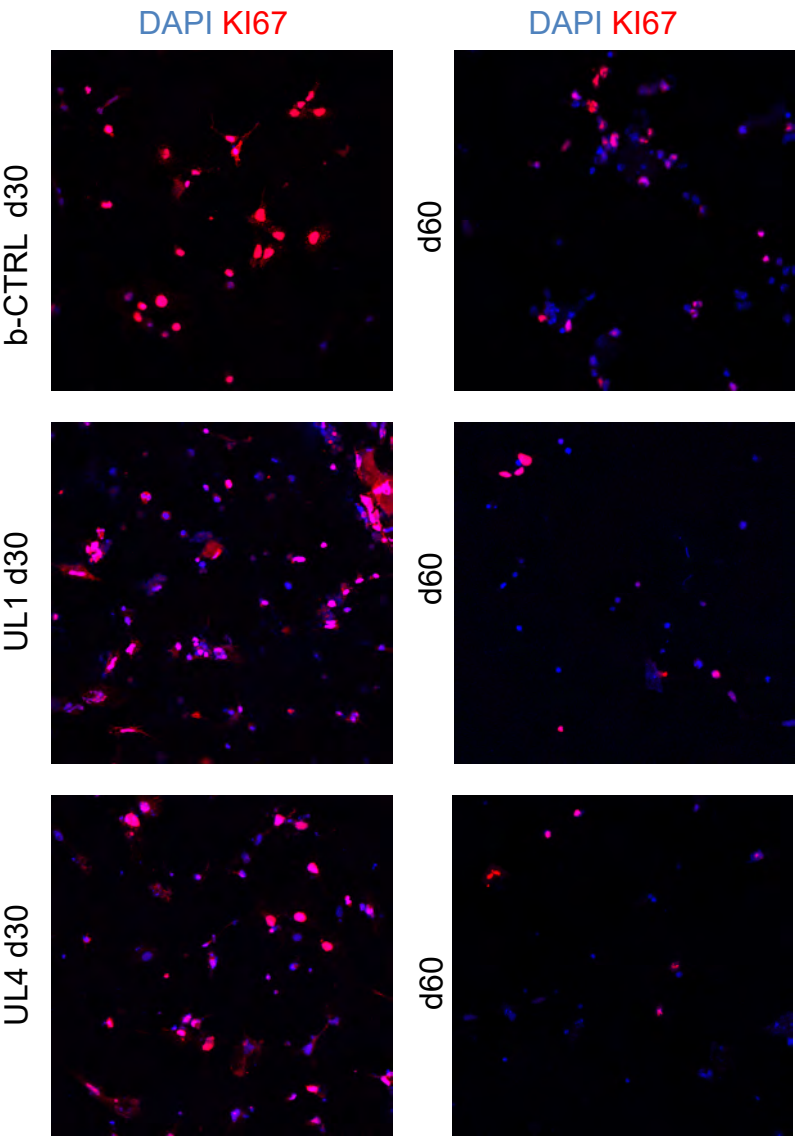

Supplement: Supplementary file 5 — Source Data for Expanded View [file EMMM-12-e11419-s011.zip › FigEV4_sourcedata.pdf]

Figure EV2

Figure EV2D

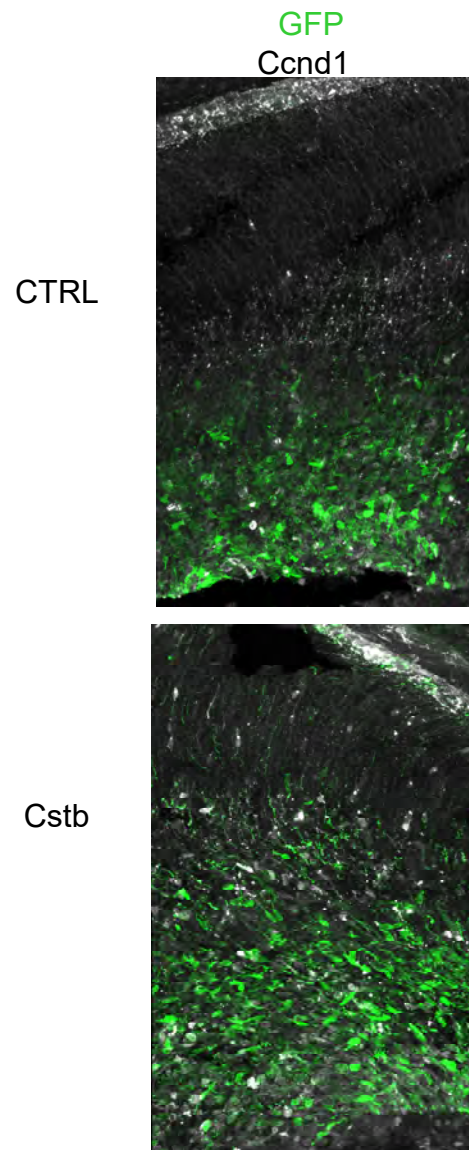

Supplement: Supplementary file 5 — Source Data for Expanded View [file EMMM-12-e11419-s011.zip › FigEV2_sourcedata.pdf]

Figure1

Figure1D

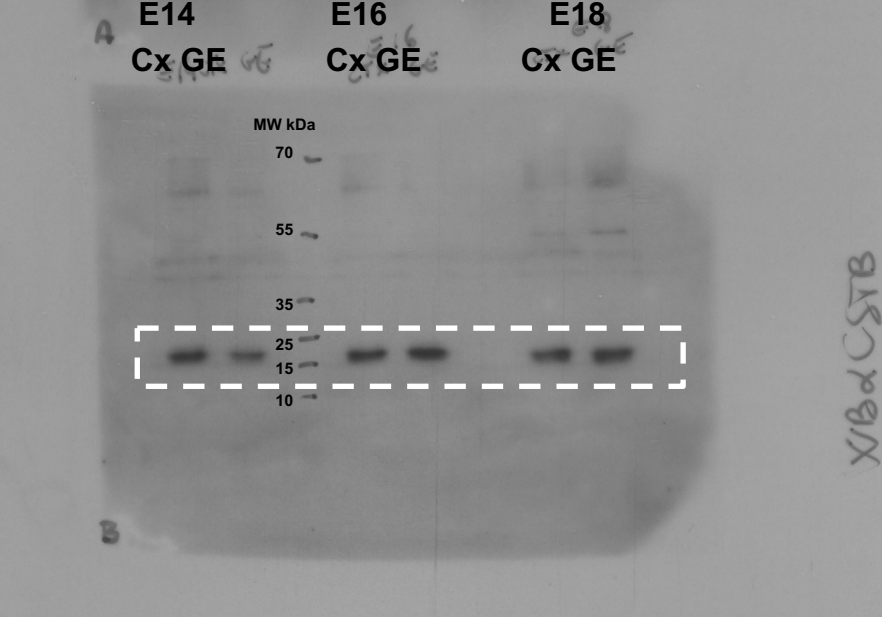

Cstb

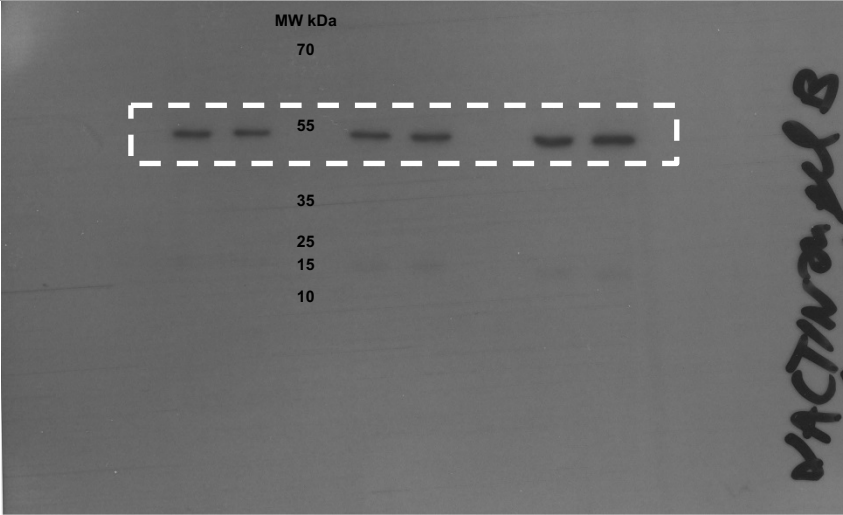

Actb

Supplement: Supplementary file 7 — Source Data for Figure 1 [file EMMM-12-e11419-s005.pdf]

Figure2

Figure2A

GFP KI67

CTRL

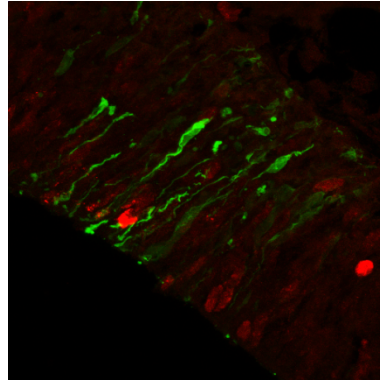

CSTB

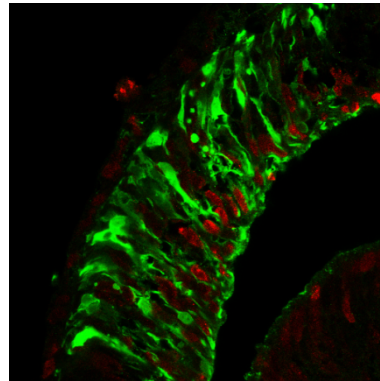

Supplement: Supplementary file 8 — Source Data for Figure 2 [file EMMM-12-e11419-s006.pdf]

Figure 3

Figure 3A

CSF E14

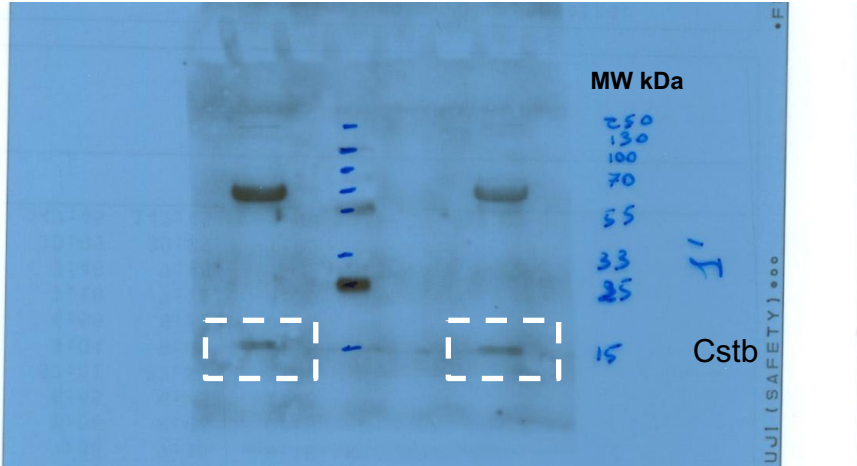

Figure 3B

Cstb in CM from E14 cortex cells

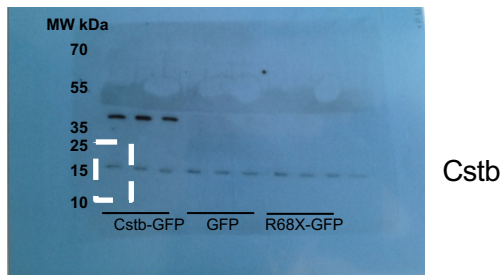

Figure 3C

Cystatin B

GAPDH

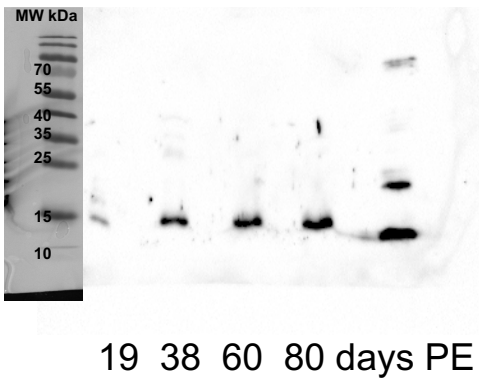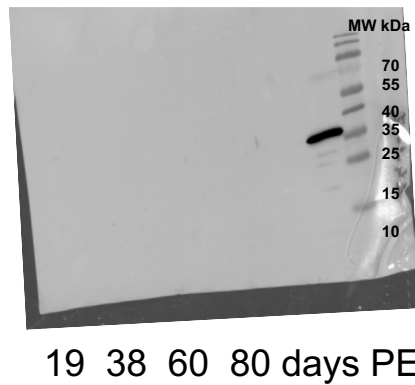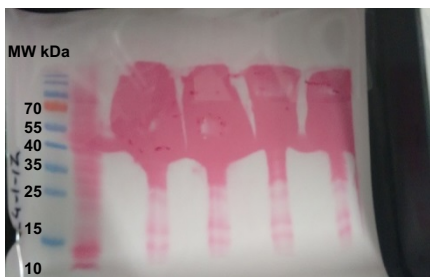

Ponceau Red

Supplement: Supplementary file 9 — Source Data for Figure 3 [file EMMM-12-e11419-s007.pdf]

Figure 4

Figure 4C

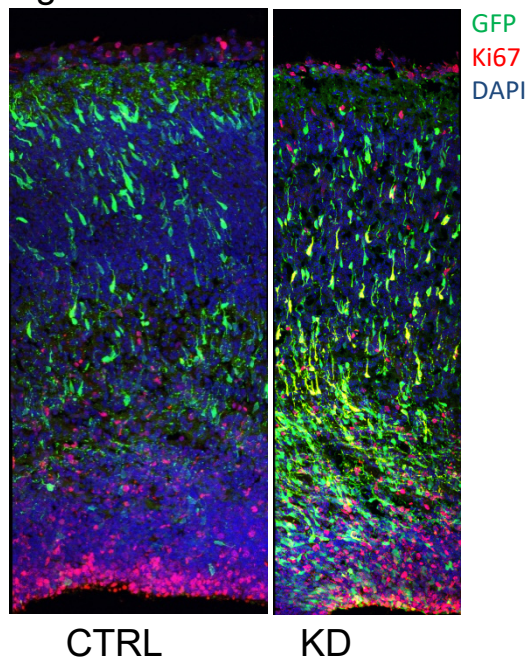

Figure 4H

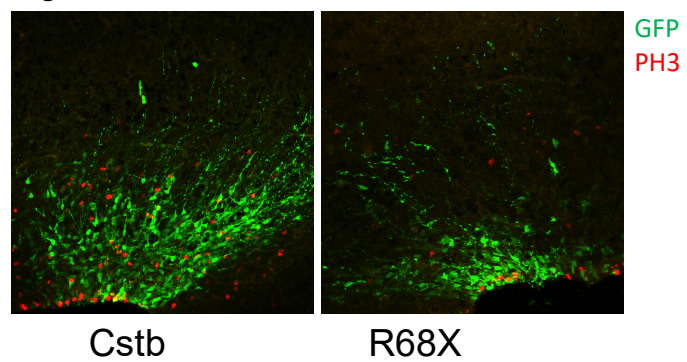

Figure 4L

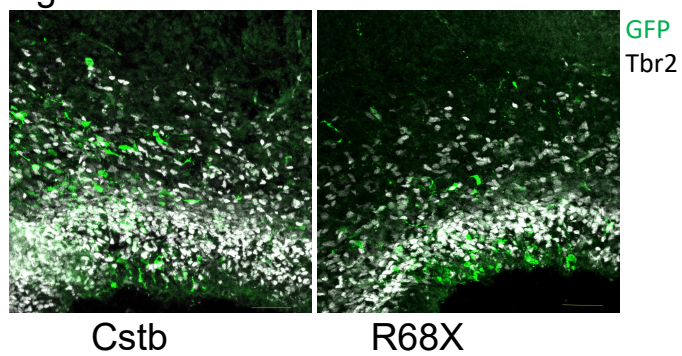

Figure 4N

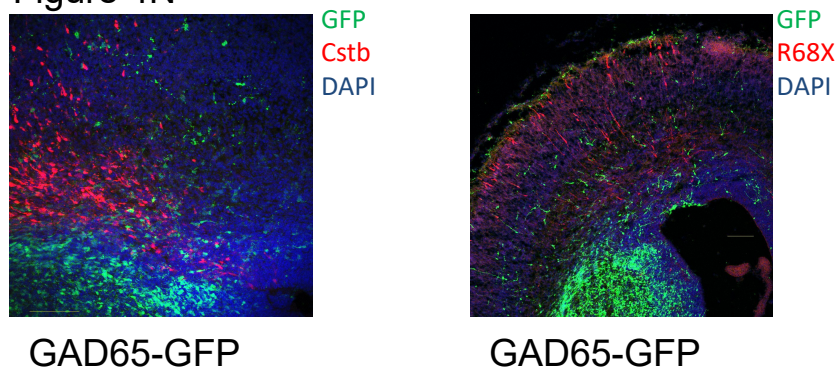

Supplement: Supplementary file 10 — Source Data for Figure 4 [file EMMM-12-e11419-s008.pdf]

Figure 5

Figure 5B

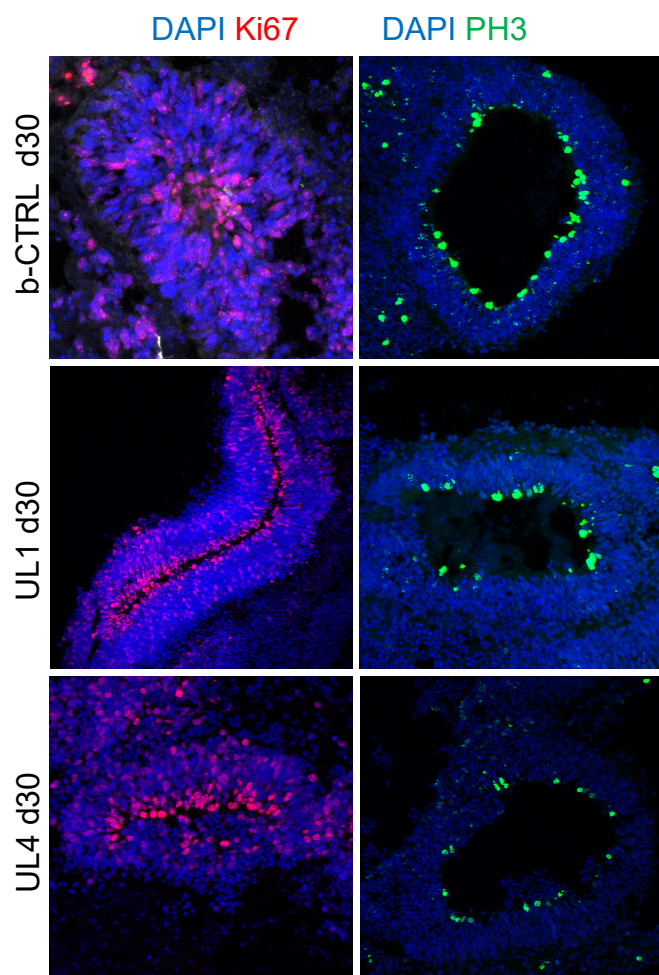

Figure 5E

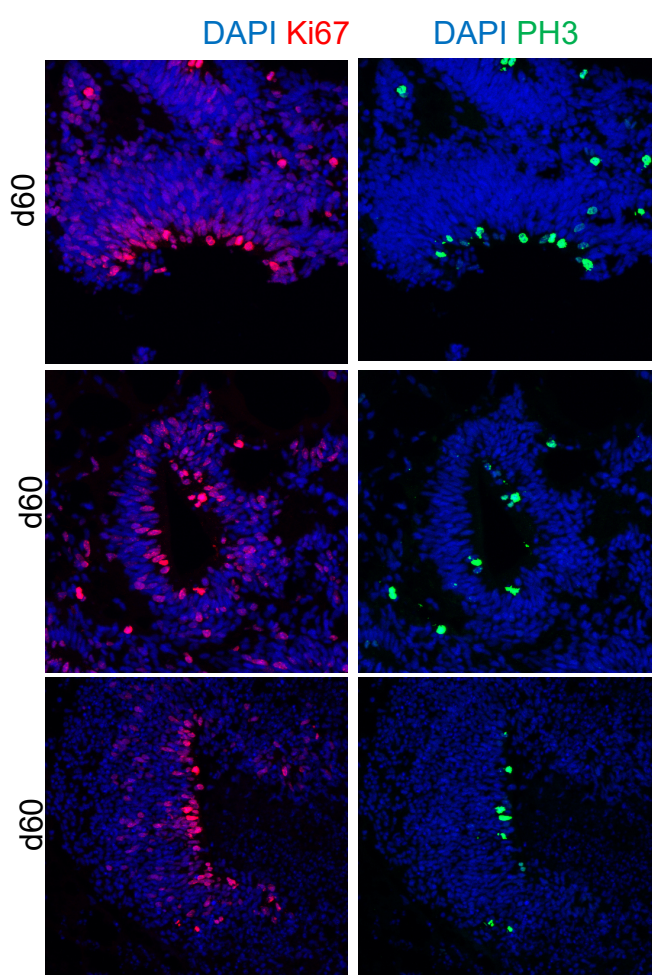

Supplement: Supplementary file 11 — Source Data for Figure 5 [file EMMM-12-e11419-s009.pdf]

Figure 6

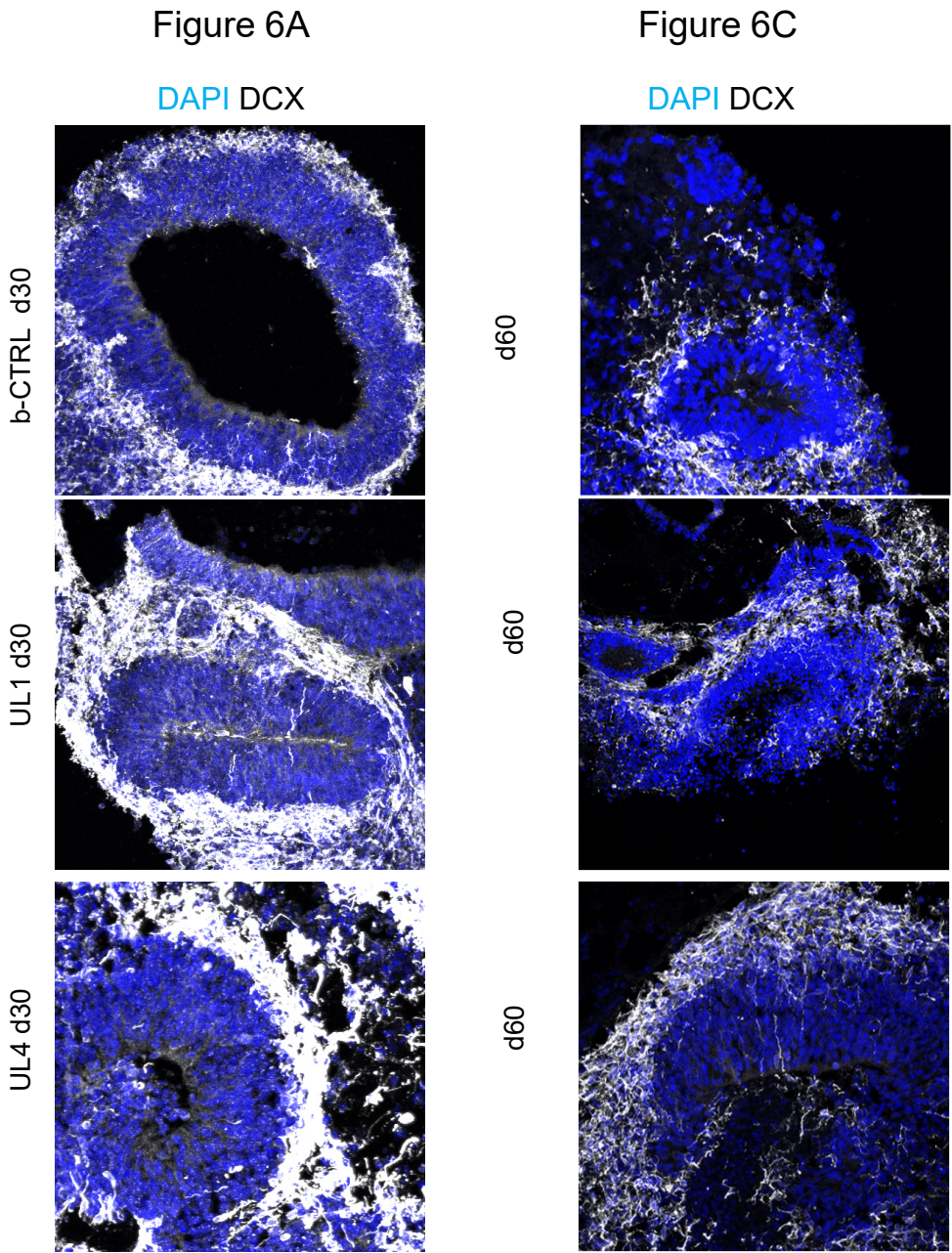

Figure 6G

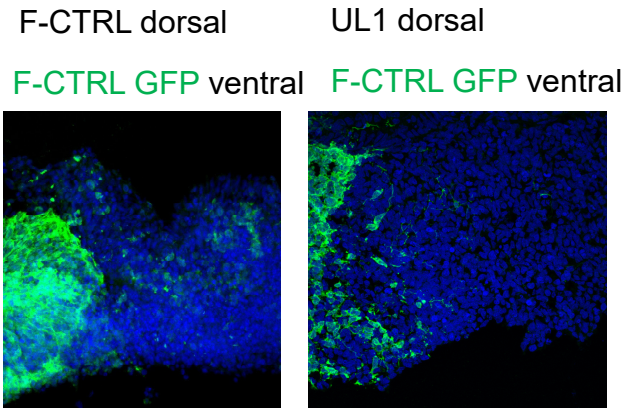

Supplement: Supplementary file 12 — Source Data for Figure 6 [file EMMM-12-e11419-s010.pdf]
